# Diagnosis and prognosis of neutrophil gelatinase-associated lipocalin for acute kidney injury with sepsis: a systematic review and meta-analysis

**DOI:** 10.1186/s13054-016-1212-x

**Published:** 2016-02-16

**Authors:** An Zhang, Ying Cai, Peng-Fei Wang, Jian-Ning Qu, Zhen-Chun Luo, Xiao-Dong Chen, Bin Huang, Yi Liu, Wen-Qi Huang, Jing Wu, Yue-Hui Yin

**Affiliations:** Department of Intensive Care Medicine, The Second Affiliated Hospital of Chongqing Medical University, Chongqing, 400010 China; Department of Cardiology, The Second Affiliated Hospital of Chongqing Medical University, Chongqing, 400010 China

**Keywords:** Neutrophil gelatinase-associated lipocalin, Acute kidney injury, Sepsis

## Abstract

**Background:**

Neutrophil gelatinase-associated lipocalin (NGAL) has been identified as an early biomarker for prediction of acute kidney injury (AKI). However, the utility of NGAL to predict the occurrence of AKI in septic patients remains controversial. We performed a systematic review and meta-analysis to evaluate the evidence on diagnosis of sepsis AKI and the prediction of other clinical outcomes.

**Method:**

The MEDLINE, EMBASE, Cochrane Library, Wanfang, and CNKI databases were systematically searched up to August 19, 2015. Quality assessment was applied by using the Quality Assessment for Studies of Diagnostic Accuracy (QUADAS-2) tool. The diagnostic performance of NGAL for the prediction of AKI in sepsis was evaluated using pooled estimates of sensitivity, specificity, likelihood ratio, and diagnostic odds ratio (DOR), as well as summary receiver operating characteristic curves (SROC).

**Results:**

Fifteen studies with a total of 1,478 patients were included in the meta-analysis. For plasma NGAL, the pooled sensitivity and specificity with corresponding 95 % confidence intervals (CI) were 0.83 (95 % CI: 0.77 − 0.88) and 0.57 (95 % CI: 0.54 − 0.61), respectively. The pooled positive likelihood ratio (PLR) was 3.10 (95 % CI: 1.57 − 6.11) and the pooled negative likelihood ratio (NLR) was 0.24 (95 % CI: 0.13 − 0.43). The pooled DOR was 14.72 (95 % CI: 6.55 − 33.10) using a random effects model. The area under the curve (AUC) for SROC to summarize diagnostic accuracy was 0.86. For urine NGAL, the pooled sensitivity, specificity, PLR, NLR, DOR, and AUC values were 0.80 (95 % CI: 0.77 − 0.83), 0.80 (95 % CI: 0.77 − 0.83), 4.42 (95 % CI: 2.84 − 6.89), 0.21 (95 % CI: 0.13 − 0.35), 24.20 (95 % CI: 9.92 − 59.05) and 0.90, respectively. Significant heterogeneity was explored as a potential source. There was no notable publication bias observed across the eligible studies. NGAL for prediction of renal replacement therapy (RRT) and mortality associated with AKI in septic patients were also evaluated.

**Conclusion:**

To a certain extent, NGAL is not only an effective predictive factor for AKI in the process of sepsis, but also shows potential predictive value for RRT and mortality. However, future trials are needed to clarify this controversial issue.

## Background

Acute kidney injury (AKI) is a widespread problem in critically ill patients, with a high and rapidly increasing incidence, particularly in the ICU, as a potentially life-threatening factor associated with significant morbidity and mortality [1, 2]. Sepsis is a well-known precipitating factor for the development of AKI, accounting for almost half of all such cases [3, 4]. The diagnosis of AKI relies on serum creatinine according to the guidelines without reflection of kidney damage. Early accurate prediction and intervention in the initial periods of AKI in sepsis play a critical role in improving the prognosis of the disease. Neutrophil gelatinase-associated lipocalin (NGAL), a member of the lipocalin superfamily that is expressed by neutrophils and various epithelial cells [5], is one of the most frequently investigated and a rapidly emerging biomarker for early prediction of different clinical settings of AKI [6]. Along with the deepening understanding, similar studies have also gradually increased the accuracy of prediction of sepsis-induced AKI. However, it remains controversial whether NGAL is a predictive biomarker of early AKI in septic patients because of the lack of corresponding statistical data. Therefore, in view of this confusion, we performed a systematic review and meta-analysis to evaluate the evidence on diagnosis of sepsis AKI to predict clinical outcomes of renal replacement therapy (RRT) and mortality.

## Methods

This systematic review was based on previous published studies, thus no ethical approval and patient consent were required.

### Study search strategy

Two investigators (P-FW and YL) systematically and independently searched the MEDLINE (via PubMed interface), EMBASE, Cochrane Library, Wanfang, and CNKI (China National Knowledge Infrastructure) databases to 19 August 2015. Also, additional studies were evaluated by reviewing the reference lists and hand-searching bibliographies of relevant articles. The literature search included the keywords and MeSH terms “neutrophil gelatinase-associated lipocalin,” “NGAL,” “sepsis,” “severe sepsis,” and “septic shock,” with no language restrictions.

### Study selection

Two of the investigators (P-FW and YL) independently determined study eligibility by reviewing each of the citations and retrieving the literature by titles or abstracts, and subsequently the full texts. Any difference in opinion regarding eligibility was resolved through consensus with an arbitrator (AZ). The studies were included in this review if they met the following set of inclusion criteria: human studies with participants ≥18 years of age; plasma/serum or urine NGAL for prediction of AKI in septic patients or prediction of RRT or mortality in septic patients with AKI (including patients of sepsis with AKI and non-AKI); included a total number of at least 40 septic patients; and the American College of Chest Physicians/Society of Critical Care Medicine (ACCP/SCCM) [7], Society of Critical Care Medicine/European Society of Intensive Care Medicine/American College of Chest Physicians/American Thoracic Society/Surgical Infection Society (SCCM/ESICM/ACCP/ATS/SIS) [8], or “Survival Sepsis Campaign 2012” [9] consensus criteria as a reference standard for defining sepsis and the Risk, Injury, Failure, Loss, End-Stage Kidney Disease (RIFLE) [10], Acute Kidney Injury Network (AKIN) [11], or Kidney Disease Improving Global Outcomes (KDIGO) [12] creatinine criteria for defining AKI. Studies were excluded if they: focused on chronic kidney disease, or renal transplantation, etc.; were duplicate articles describing the same study; were articles not based on original studies, such as reviews, commentaries, conference abstracts, letters, supplementary issues, poster presentations, or editorials; and contained insufficient information.

### Data extraction and quality assessment

Two reviewers (P-FW and YL) independently extracted prespecified data elements from each trial, including the request for documentation and recalculation of the following variables: first author, year of publication, study location, study design, admission setting, definitions of AKI and sepsis, sampling time, and method for NGAL measurement using a standardized data extraction form. Also, the diagnostic test performed, the area under the curve (AUC), optimal cutoff thresholds, sensitivity, specificity, and number of AKI and non-AKI septic patients were obtained from the enrolled articles. Absolute data of true-positive (TP), false-positive (FP), true-negative (TN) and false-negative (FN) rates or equivalent data were provided or could be calculated for constructing 2 × 2 contingency tables. If only the AUC data without a 2 × 2 table data were included, we contacted the corresponding authors by email and asked whether they were willing to share their unpublished data in the articles for inclusion in the present study. If no reply was received, the study was excluded from the meta-analysis and included in the descriptive analysis only. We attempted to extract data to evaluate the prediction of RRT and mortality of AKI among septic patients. However, since these data were rarely reported in the studies, we only included a simple description in the subsequent analysis.

Two reviewers (J-NQ and W-QH) independently assessed the methodological quality of the studies using the Quality Assessment of Diagnostic Accuracy Studies 2 (QUADAS-2) tool for quality assessment and accuracy of diagnostic studies [13]. The QUADAS-2 tool is based on four key domains: patient selection, index test, reference standard, and flow and timing. “Risk of bias” and “concerns regarding applicability” were evaluated for all four domains and the first three domains, respectively, with each item judged as “yes,” “no,” or “unclear.” To judge the “risk of bias,” if the answers to all signaling questions were “yes” in a domain, the domain was judged as having a low risk of bias. Any signaling question that was answered “no” indicated a high risk of bias. The judgment principle of “applicability” was the same as the bias section, but without signaling questions.

### Statistical analysis

TP, TN, FP, and FN rates for each test in every study were analyzed using Meta-DiSc (version 1.4) software, as described elsewhere [14], to assess the sensitivity, specificity, positive likelihood ratio (PLR), negative likelihood ratio (NLR), and diagnostic odds ratio (DOR) for each included study. The summary measures were calculated using a random effects model (DerSimonian and Laird method) for high heterogeneity, otherwise a fixed effect model was chosen (Mantel − Haenszel method). Forest plots of accuracy indexes were also constructed. To describe the relationship between test sensitivity and specificity, a summary receiver operating characteristics (SROC) curve was constructed based on TP and FP rates. The AUC was calculated as an overall summary index to measure the diagnostic performance for the prediction of AKI in septic patients by NGAL, which has been defined as a useful risk predictor when AUC ≥0.70 [15]. The Q* index is defined as the closest point to the idea top-left corner on the SROC curve where sensitivity equals specificity [16]. A threshold effect is an important cause of heterogeneity in diagnostic testing that can be observed as a typical pattern of a “shoulder-arm” shape in the SROC plane and confirmed by the Spearman correlation coefficient and probability (*P*) value between the logit of sensitivity and logit of 1 – specificity. *P* <0.05 indicated the existence of a threshold effect [17]. Heterogeneity caused by nonthreshold effects was assessed by applying the Cochran’s Q test and the *I*^2^ index. For the former, it is considered that more such studies will be homogeneous when the statistical significance is set at *P* >0.10, according to a chi-squared distribution with *k* – 1 degrees of freedom [18]. The *I*^2^ index was used to measure the degree of heterogeneity between multiple studies. *I*^2^ values <25 %, of 25–50 %, and >50 % indicated modest, moderate, and substantial heterogeneity, respectively. Likelihood ratios state how many times more likely particular test results would be accurate for patients with disease than for subjects without disease [19].

In order to eliminate factors that influence heterogeneity, sensitivity analysis was conducted to examine the stability by omitting one study at a time. Meta-regression and subgroup analyses were also performed to identify factors that contributed to heterogeneity. The heterogeneity test, assessment of threshold effect, diagnostic performance, as well as meta-regression and subgroup analyses were also performed using Meta-DiSc (version 1.4) software [14]. In addition, publication bias was assessed with the Deeks test using Stata (version 12.0) statistical software (STATA Corporation, College Station, TX, USA) when >10 studies were evaluated [20].

## Results

### Study characteristics and quality assessment

**Fig. 1:**
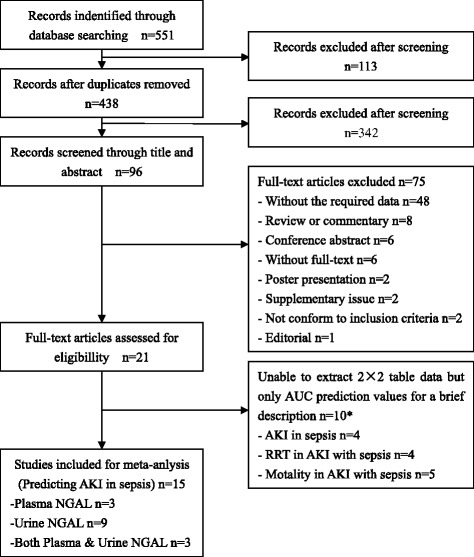
Study flow diagram. *Several distinct but valid data were extracted from one single literature item (a total number of 10 literatures), in which multiple endpoints in clinical trials were incorporated. *AKI* acute kidney injury, *AUC* area under the curve, *NGAL* neutrophil gelatinase-associated lipocalin, *RRT* renal replacement therapy

The initial search identified 551 relevant articles from various databases, of which 113 were excluded because of duplication. Of the remaining 438 studies, 342 were excluded after screening the titles and abstracts. After screening the full texts, 75 studies were excluded in accordance with the eligibility criteria. Eligibility of the remaining 21 studies was assessed. Among these, 15 studies [21–35] that included 1478 patients published between 2010 and 2015 were included for meta-analysis for prediction of AKI in septic patients. The included studies were geographically diverse: four studies were conducted in Europe, two in America, and the remaining studies were of Asia origin. Of the 21 studies, 10 included descriptive analysis of the prediction AKI in septic patients and RRT or mortality in sepsis-induced AKI without 2 × 2 table data. A flowchart depicting the literature screening method is shown in Fig. 1. The main features and detailed information of each study included for the meta-analysis are presented in Table 1, and the quality assessment of the included 15 studies is presented in Table 2.

**Table 1:** Characteristics of included studies for NGAL to predict AKI in septic patients

Study	Location	Design	Setting	AKI definition	Sepsis definition	Source	AKI/ sepsis (*n*)	Sampling time (hours)	Storage (°C)	NGAL assay	NGAL test kits
Aydogdu et al. [21]	Turkey	PC	ICU	RIFLE	SCCM/ESICM/ACCP/ATS/SIS	Urine	63/129	NR	−80	ELISA	Biovendor (Brno, Southern Moravia, Czech Republic)
Camou et al. [31] (ClinicalTrials.gov NCT01122225)	France	PC	ICU	RIFLE or AKIN	SCCM/ESICM/ACCP/ATS/SIS	Plasma	43/50	Admission	NR	ELISA	Triage (Biosite Inc., San Diego, CA, USA)
de Geus et al. [34]	Netherlands	PC	ICU	AKIN	ACCP/ SCCM	Plasma	50/75	Admission	−80	ELISA	Triage (Biosite Inc.)
Fan et al. [30]	China	PC	ICU	RIFLE	SCCM/ESICM/ACCP/ATS/SIS	Urine	58/126	Peak	NR	RIA	–
Hjortrup et al. [29] (ClinicalTrials.gov NCT00962156)	Denmark	PC	ICU	KDIGO	ACCP/SCCM	Plasma	31/124	Admission	−80	PETIA	BioProto Diagnostics A/S (Gentofte, Denmark)
						Urine	25/100		−24		
Li and Xu [22]	China	PC	ICU	AKIN	ACCP/SCCM	Urine	17/74	24	−20	ELISA	R&D Systems (Minneapolis, MN, USA)
Martensson et al. [33]	Sweden	NR	ICU	RIFLE or AKIN	ACCP/SCCM	Plasma	18/45	12	NR	RIA	–
						Urine	18/45		NR	RIA	–
Niu et al. [23]	China	PC	ER	AKIN	SCCM/ESICM/ACCP/ATS/SIS	Urine	26/60	12	−80	ELISA	Hycult Biotech (Uden, North Brabant, The Netherlands)
Shapiro et al. [24]	USA	PC	ER	RIFLE	ACCP/SCCM	Plasma	24/66	Admission	−70	ELISA	Triage (Biosite Inc.)
Si et al. [32]	Brazil	PC	ER	AKIN	SSC	Urine	47/168	Admission	−80	ELISA	NR
Wang et al. [25]	China	NR	ICU	KDIGO	ACCP/SCCM	Urine	33/87	48^a^	−80	ELISA	R&D Systems
Xing et al. [26]	China	NR	ICU	AKIN	SCCM/ESICM/ACCP/ATS/SIS	Plasma	35/73	NR	−80	ELISA	R&D Systems
						Urine	35/73		−20	ELISA	R&D Systems
Yan et al. [27]	China	NR	ICU	AKIN	ACCP/SCCM	Urine	57/112	2	−80	ELISA	R&D Systems
Yan and Zang [28]	China	PC	ICU	AKIN	SCCM/ESICM/ACCP/ATS/SIS	Urine	44/141	8	−80	ELISA	R&D Systems
Zhou et al. [35]	China	NR	ICU	AKIN	SCCM/ESICM/ACCP/ATS/SIS	Urine	46/148	8	−80	ELISA	R&D Systems

^a^Forty-eight hours before AKI

*ACCP/SCCM* American College of Chest Physicians/Society of Critical Care Medicine, *AKI* acute kidney injury, *AKIN* Acute Kidney Injury Network, *ELISA* enzyme-linked immunosorbent assay, *ER* emergency room, *KDIGO* Kidney Disease: Improving Global Outcomes, *NGAL* neutrophil gelatinase-associated lipocalin, *NR* not reported, *PC* prospective cohort, *PETIA* particle-enhanced turbidimetric immunoassay, *RIA* radioimmunoassay, *RIFLE* Risk, Injury, Failure, Loss, End-Stage Kidney Disease, *SSC* Survival Sepsis Campaign, *SCCM/ESICM/ACCP/ATS/SIS* Society of Critical Care Medicine/European Society of Intensive Care Medicine/American College of Chest Physicians/American Thoracic Society/Surgical Infection Society

**Table 2:**
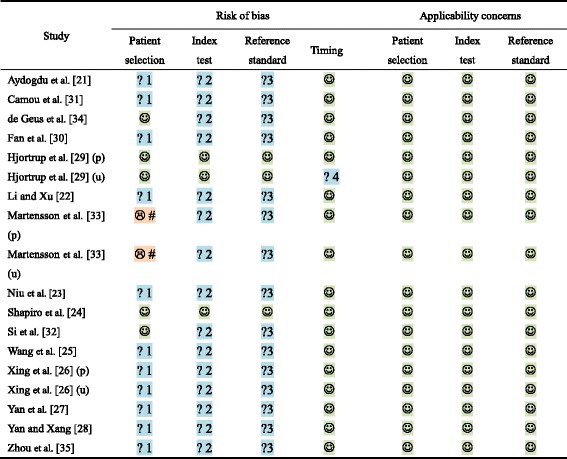
Quality assessment of included eligible studies using QUADAS-2

low risk,  high risk,  unclear risk, *p* plasma, *u* urine

*1* unknown whether a consecutive or random sample of patients enrolled, *2* unknown whether the index test results interpreted without knowledge of the results of the reference standard or unknown whether the threshold was prespecified, *3* unclear whether the reference standard results were interpreted without knowledge of the results of the index test, *4* unknown whether all patients were included in the analysis, *#* unknown whether enrolling a consecutive or random sample without introduction

*QUADAS-2* Quality Assessment of Diagnostic Accuracy Studies 2

### Diagnostic performance

#### NGAL for prediction of AKI in septic patients

##### Plasma NGAL

**Fig. 2:**
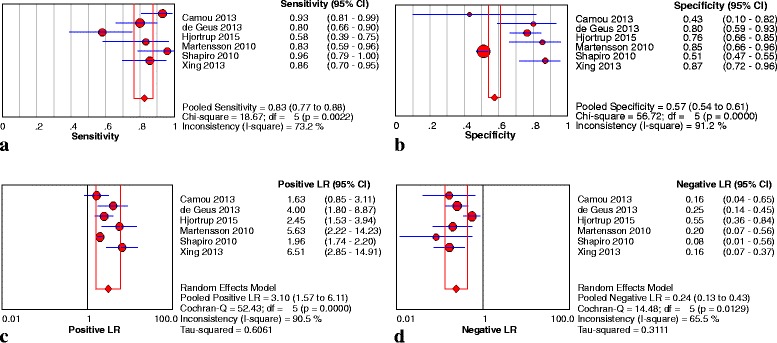
Sensitivity **a**, specificity **b**, PLR **c** and NLR **d** of plasma NGAL for prediction of AKI in sepsis. *CI* confidence interval, *LR* likelihood ratio

Six studies [24, 26, 29, 31, 33, 34] enrolling 433 patients investigated the predictive value of plasma NGAL as a biomarker of AKI in septic patients. The key results of these studies are summarized in Table 3. The pooled sensitivity and specificity were 0.83 (95 % confidence interval (CI): 0.77–0.88) and 0.57 (95 % CI: 0.54 − 0.61), respectively. The pooled PLR was 3.10 (95 % CI: 1.57 − 6.11) and the pooled NLR was 0.24 (95 % CI: 0.13 − 0.43) (Fig. 2). The pooled DOR was 14.72 (95 % CI: 6.55 − 33.10) using a random effects model. We performed SROC to summarize the diagnosis accuracy: the AUC was 0.86 (standard error (SE) = 0.04) with Q* of 0.79 (SE = 0.04), indicating good, but not excellent, diagnostic accuracy (see Fig. 4).

**Table 3:** Diagnostic value of NGAL to predict AKI in septic patients in individual studies

Study	AUC	95 % CI	Cutoff value	Sensitivity (%)	Specificity (%)	Number of patients			
						TP rate	FP rate	FN rate	TN rate
Aydogdu et al. [21]	0.80	NR	29.5 ng/ml	0.88	0.73	55	18	8	48
Camou et al. [31]	0.90	NR	150 ng/ml	0.93	0.44	40	4	3	3
de Geus et al. [34]	0.80	0.69–0.88	304 ng/ml	0.8	0.80	40	5	10	20
Fan et al. [30]	0.86	0.81-0.93	NR	0.89	0.74	52	18	6	50
Hjortrup et al. [29] (p)	0.66	0.54–0.77	NR	0.58	0.76	18	22	13	71
Hjortrup et al. [29] (u)	0.71	0.59–0.82	NR	0.56	0.72	14	21	11	54
Li and Xu [22]	0.94	0.68-0.97	50 μg/l	0.94	0.88	16	7	1	50
Martensson et al. [33] (p)	0.85	0.39-0.94	>120 ng/ml	0.83	0.86	15	4	3	23
Martensson et al. [33] (u)	0.86	0.68-1.00	>68 ng/mg.scr	0.71	1.00	13	0	5	27
Niu et al. [23]	0.91	NR	52 μg/g · scr	0.88	0.87	23	5	3	29
Shapiro et al. [24]	0.82	0.76-0.88	NR	0.96	0.51	23	312	1	325
Si et al. [32]	0.73	0.64-0.82	3.36 ng/ml	0.63	0.46	76	25	45	22
Wang et al. [25]	0.81	0.71-0.91	150 ng/ml	0.79	0.90	26	5	7	49
Xing et al. [26] (p)	0.86	0.77-0.94	92.5 ng/ml	0.85	0.87	30	5	5	33
Xing et al. [26] (u)	0.93	0.88-0.93	118.5 ng/ml	0.93	0.89	32	4	3	34
Yan et al. [27]	0.93	0.88-0.98	65 μg/l	0.95	0.86	54	8	3	47
Yan and Xang [28]	0.86	0.70-0.96	90 μg/l	0.87	0.86	38	14	6	83
Zhou et al. [35]	0.80	0.7 l-0.93	85 ng/l	0.78	0.80	36	20	10	82

*AKI* acute kidney injury, *AUC* area under the curve, *CI* confidence interval, *FP* false-positive, *FN* false-negative, *NGAL* neutrophil gelatinase-associated lipocalin, *NR* not reported, *p* plasma, *TP* true-positive, *TN* true-negative, *u* urine

Four studies lacked 2 × 2 contingency table data for the meta-analysis, but included AUC prediction values for a brief description. In an observational study of septic patients admitted to the emergency room (ER) [36], the AUC of serum NGAL for prediction of AKI in septic patients was 0.90 (95 % CI: 0.85–0.94), greater than that of procalcitonin. In another observational retrospective study [37], the diagnostic accuracy of plasma NGAL for prediction of AKI among the 101 consecutive investigated septic patients admitted to the ER had an AUC of 0.80. Yamashita et al*.* [38] reported predominantly high AUCs of 0.94 (95 % CI: 0.88–0.97) and 0.92 (95 % CI: 0.84–0.96) for plasma NGAL to predict AKI and severe AKI in septic patients, respectively. When NGAL was combined with the clinical model, the AUC was 0.89 (95 % CI: 0.77–0.95), slightly better than with only a clinical model (0.87; 95 % CI: 0.76–0.94) for detecting severe AKI in septic patients. Dai et al*.* [39] showed that the AUC of plasma NGAL was 0.83 (95 % CI: 0.74 − 0.92), indicating that NGAL was a good indicator of the occurrence of AKI at 24 hours in septic patients.

There was no notable threshold effect in the six studies included in the meta-analysis. Given the small sample size of most of the included studies, we did not conduct meta-regression or subgroup analyses, although we performed sensitivity analysis to eliminate factors that influence heterogeneity by omitting each one study at a time. The pooled AUC estimated by the remaining studies did not change significantly, with the exception of one of the studies (Table 4). After removing that study [29], sensitivity was 0.87 (95 % CI: 0.81–0.92), specificity was 0.55 (95 % CI: 0.51–0.59), PLR was 3.33 (95 % CI: 1.23–9.03), NLR was 0.20 (95 % CI: 0.13–0.30), DOR was 22.13 (95 % CI: 11.20 − 43.73), and the AUC reached 0.90. These changes might be related to the study that was a substudy of the 6S trial (6S—Scandinavian Starch for Severe Sepsis/Septic Shock trial) [40]. The 6S trial compared the effect of hydroxyethyl starch 130/0.4 with Ringer’s acetate or Ringer’s acetate alone on kidney failure in patients with severe sepsis. The differences in fluid resuscitation might have led to the potential heterogeneity. With the exception of this study, no other individual study significantly influenced the meta-analysis results, thereby indicating a certain credibility of the outcomes.

**Table 4:** Pooled AUC and 95 % CI after omitting each trial in the meta-analysis (sensitivity analysis)

Study	Sensitivity	Specificity	Positive likelihood ratio	Negative likelihood ratio	Diagnostic odds ratio	AUC
	(95 % CI)	(95 % CI)	(95 % CI)	(95 % CI)	(95 % CI)	
Total	0.83 (0.77–0.88)	0.57 (0.54–0.61)	3.10 (1.57–6.11)	0.24 (0.13–0.43)	14.72 (6.55–33.10)	0.86
Camou et al. [31]	0.80 ( 0.73–0.86)	0.58 (0.54–0.61)	3.59 (1.45–8.88)	0.25 (0.13–0.48)	15.93 (6.21–40.85)	0.87
de Geus et al. [34]	0.83 (0.77–0.89)	0.57 (0.53–0.60)	2.94 (1.45–5.93)	0.22 (0.10–0.50)	14.98 (5.37–41.80)	0.86
Hjortrup et al. [29]	0.87 (0.81–0.92)	0.55 (0.51–0.59)	3.33 (1.23–9.03)	0.20 (0.13–0.30)	22.13 (11.20–43.73)	0.90
Martensson et al. [33]	0.83 (0.76–0.88)	0.57 (0.53–0.60)	2.78 (1.43–5.41)	0.24 (0.12–0.48)	13.27 (5.35–32.93)	0.85
Shapiro et al. [24]	0.81 (0.74–0.86)	0.79 (0.72–0.85)	3.34 (1.98–5.64)	0.26 (0.15–0.47)	14.03 (5.66–34.77)	0.86
Xing et al. [26]	0.82 (0.75–0.87)	0.56 (0.52–0.60)	2.63 (1.51–4.59)	0.26 (0.14–0.50)	11.38 (5.11–25.31)	0.83

*AUC* area under the curve, *CI* confidence interval

##### Urine NGAL

**Fig. 3:**
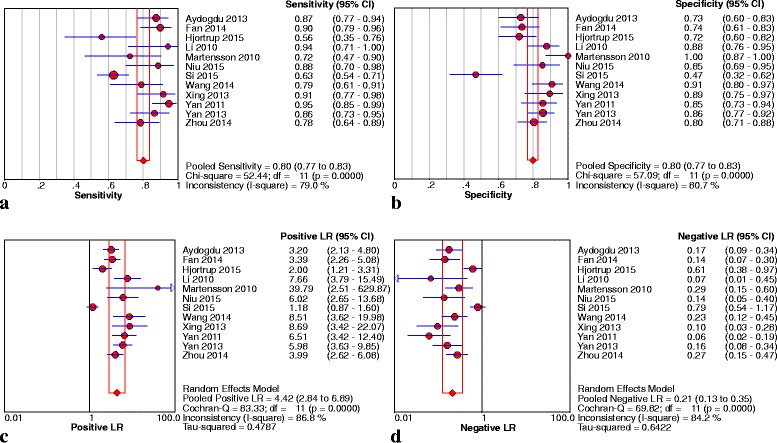
Sensitivity **a**, specificity **b**, PLR **c** and NLR **d** of urine NGAL for prediction of AKI in sepsis. *CI* confidence interval, *LR* likelihood ratio

**Fig. 4:**
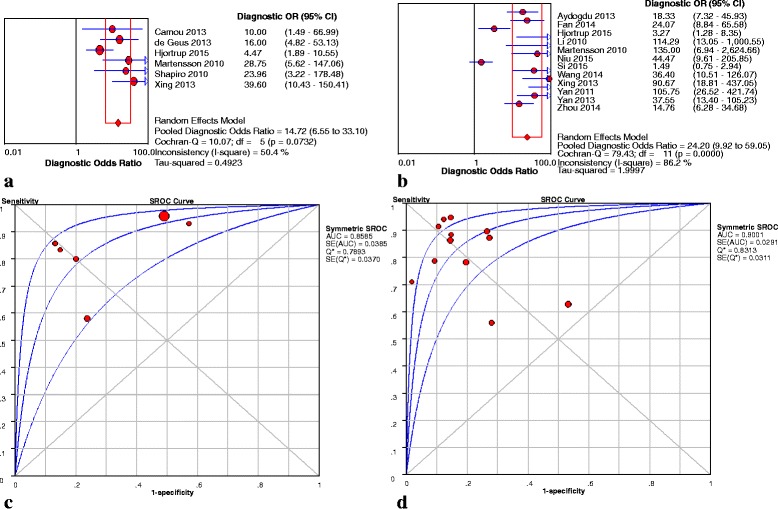
DOR and SROC curves of plasma and urine NGAL for prediction of  septic AKI. **a** DOR of plasma NGAL for prediction of  septic AKI. **b** DOR of urine NGAL for prediction of  septic AKI. **c** SROC curve of plasma NGAL for prediction of  septic AKI. **d** SROC curve of urine NGAL for prediction of  septic AKI. *AUC* area under the curve, *CI* confidence interval, *SROC* summary receiver operating characteristic, *OR* odds ratio, *SE* standard error

For urine NGAL, 12 studies [21–23, 25–30, 32, 33, 35] enrolling 1263 persons were included in the pooled diagnostic assessment of performance. The key results of these studies are summarized in Table 3. The pooled sensitivity and specificity were 0.80 (95 % CI: 0.77 − 0.83) and 0.80 (95 % CI: 0.77 − 0.83), respectively. The pooled PLR was 4.42 (95 % CI: 2.84 − 6.89) and the pooled NLR was 0.21 (95 % CI: 0.13 − 0.35) (Fig. 3). The pooled DOR was 24.20 (95 % CI: 9.92 − 59.05). The AUC was 0.90 (SE = 0.02) with Q* of 0.84 (SE = 0.02) (Fig. 4). Meanwhile, Yamashita et al. and Dai et al*.* also reported the usefulness of urine NGAL to predict septic AKI with AUCs of 0.84 (95 % CI: 0.72 − 0.91) and 0.88 (95 % CI: 0.79–0.95), respectively [38, 39].

**Fig. 5:**
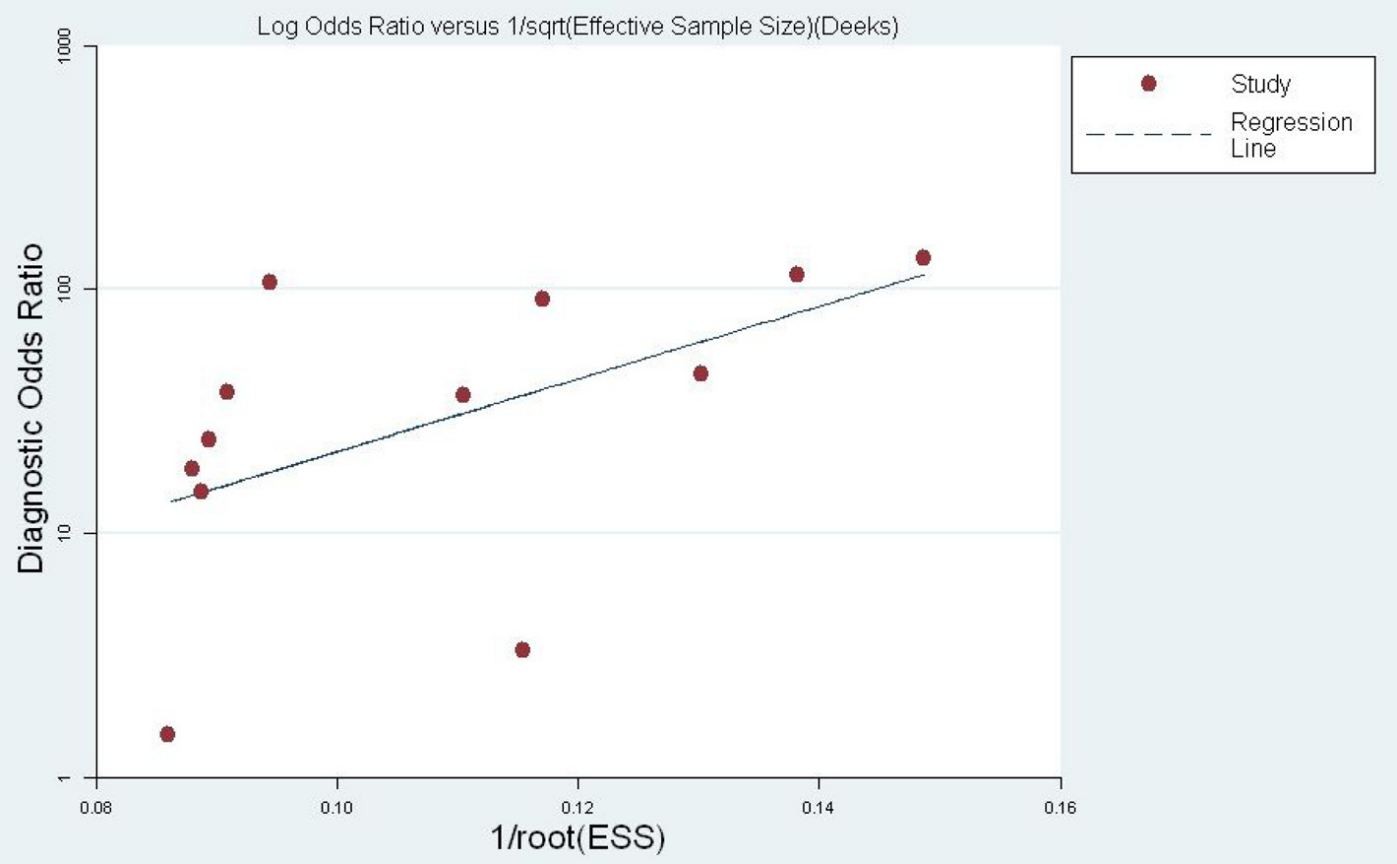
Deeks Funnel plot assessment of potential publication bias. Each solid rectangle represents an eligible study. *ESS* effective sample size

Considering the heterogeneity across these 12 eligible studies and exclusion of the threshold effect, we performed subgroup analysis between different study characteristics. The admission setting (ICU or ER), study design (prospective or nonprospective), number of cases (*n* ≥100 or *n* <100), NGAL test method (enzyme-linked immunosorbent assay (ELISA) or non-ELISA), location of the subjects (Asia or others), and publication language (English or Chinese) were hypothesized as possible factors influencing heterogeneity. The results of the subgroup analysis are presented in Table 5. Univariate meta-regression was also performed with these factors to explore the sources of heterogeneity. However, causes of heterogeneity could not be explained with *P* <0.05 (Table 5). In addition, the Deeks funnel plot indicated the absence of notable publication bias (*P* = 0.132) (Fig. 5).

**Table 5:** Possible sources of heterogeneity of meta-analysis (results of subgroup analysis and meta-regression analysis)

Variance		Number	Sensitivity	Specificity	Positive likelihood ratio	Negative likelihood ratio	Diagnostic odds ratio	AUC	Coeff.	SE	*P* value	RDOR (95 % CI)
			(95 % CI)	(95 % CI)	(95 % CI)	(95 % CI)	(95 % CI)					
Setting	ICU	10	0.85 (0.81–0.88)	0.82 (0.79–0.85)	4.73 (3.41–6.55)	0.19 (0.12–0.31)	29.05 (14.29–59.02)	0.91	–0.96	1.16	0.45	0.38 (0.02–9.57)
	ER	2	0.67 (0.59–0.75)	0.63 (0.52–0.73)	2.56 (0.48–13.65)	0.35 (0.05–2.34)	7.57 (0.27–216.18)	–				
Design	Prospective	7	0.77 (0.73–0.82)	0.76 (0.71–0.80)	3.41 (1.98–5.85)	0.24 (0.11–0.50)	15.00 (4.58–49.14)	0.84	0.16	0.80	0.85	1.18 (0.13–10.85)
	Nonprospective	5	0.85 (0.79–0.90)	0.87 (0.82–0.90)	6.40 (3.94–10.40)	0.19 (0.11–0.32)	45.65 (18.15–114.83)	0.94				
Number of cases	≥100	7	0.87 (0.74–0.82)	0.76 (0.72–0.79)	3.20 (1.95–5.24)	0.24 (0.12–0.49)	13.62 (4.53–40.98)	0.82	1.33	0.84	0.19	3.77 (0.37–38.63)
	<100	5	0.85 (0.78–0.91)	0.90 (0.85–0.94)	7.85(5.24–11.74)	0.18 (0.11–0.30)	58.18 (27.63–122.49)	0.95				
NGAL test method	ELISA	9	0.81 (0.77–0.84)	0.81 (0.77–0.84)	4.79 (2.76–8.32)	0.18 (0.09–0.35)	28.07 (9.47–83.18)	0.91	0.51	1.01	0.64	1.66 (0.10–26.97)
	Non-ELISA	3	0.78 (0.69–0.86)	0.77 (0.70–0.83)	3.16 (1.46–6.82)	0.31 (0.12–0.76)	15.56 (2.42–100.09)	0.86				
Location	Asia	9	0.88 (0.84–0.91)	0.83 (0.79–0.86)	5.01 (3.87–6.49)	0.17 (0.12–0.22)	33.05 (20.72–52.72)	0.92	–1.23	1.30	0.40	0.29 (0.01–10.88)
	Others	3	0.63 (0.55–0.70)	0.69 (0.61–0.76)	1.99 (0.83–4.76)	0.56 (0.34–0.93)	4.34 (0.96–19.62)	0.64				
Language	English	5	0.74 (0.68–0.79)	0.71 (0.65–0.76)	2.51 (1.38–4.57)	0.34 (0.17–0.69)	9.36 (2.40–36.54)	0.83	0.60	1.04	0.59	1.82 (0.10–32.62.)
	Chinese	7	0.87 (0.83–0.91)	0.86 (0.82–0.89)	5.77 (4.56–7.30)	0.16 (0.10–0.24)	41.64 (22.82–75.97)	0.93				

*AUC* area under the curve, *CI* confidence interval, *Coeff*. coefficient, *ELISA* enzyme-linked immunosorbent assay, *ER* emergency room, *NGAL* neutrophil gelatinase-associated lipocalin *SE* Standard error, *RDOR* relative diagnostic odds ratio

### NGAL for prediction of RRT in septic patients with AKI

A recent study reported that plasma and urine NGAL had relatively low predictive values for use of RRT in ICU patients with severe sepsis and, even excluding patients with chronic kidney disease, the AUCs were 0.73 (95 % CI: 0.61–0.85; *P* = 0.64) and 0.68 (95 % CI: 0.53–0.83; *P* = 0.52), respectively [29]. In another study enrolling 126 septic patients, 23 of 58 patients with septic AKI received RRT. The peak urine NGAL was higher in patients receiving hemodialysis compared with those not receiving hemodialysis (median, 456 vs. 341 ng/ml, respectively; *P* <0.0001). The AUC of the peak urine NGAL for prediction of hemodialysis was 0.77 (95 % CI: 0.64–0.83) with a cutoff level of 494 ng/ml and the sensitivity and specificity were 0.89 and 0.71, respectively [30]. The results of a subgroup study of septic AKI patients with community-acquired pneumonia who met the RIFLE-F criteria found that plasma NGAL was a poor predictor for the requirement of RRT (AUC 0.62, 95 % CI: 0.45 − 0.81) [41]. In 50 critically ill adults with septic shock, 86 % had AKI and 30 % required RRT during their ICU stay. The AUC of plasma NGAL for predicting RRT in septic AKI patients was 0.80, with an optimal cutoff level of 348 ng/ml (sensitivity, 0.93; specificity, 0.68) [31].

### NGAL for prediction of mortality in septic patients with AKI

In a prospective observational study enrolling 92 septic patients with AKI, the AUC of urine NGAL for the prediction of 180-day mortality was 0.76 (95 % CI: 0.66–0.86; *P* = 0.000), with an Acute Physiology and Chronic Health Evaluation (APACHE) II score of 0.81 (95 % CI: 0.72–0.90; *P* = 0.00), higher than for serum creatinine (0.64; 95 % CI: 0.52–0.76; *P* = 0.022). The AUCs of urine NGAL and APACHE II scores were consistent with the results of multivariate Cox regression analysis, showing that urine NGAL (*P* = 0.014) and APACHE II score (*P* = 0.015) were independent predictors of 180-day mortality [42].

For severe sepsis, plasma and urine NGAL had poor predictive values for 90-day mortality, and when combined with plasma creatinine the AUCs were 0.55 (95 % CI: 0.47 − 0.63) and 0.61 (95 % CI: 0.42 − 0.58), respectively, with optimal cutoff values of 641 and 1687 ng/ml, respectively. There were no notable differences in these AUCs when compared with plasma creatinine alone (AUC = 0.50) [29]. In a set of specific groups of septic AKI patients with community-acquired pneumonia who met the RIFLE-F criteria, the AUC of plasma NGAL for prediction of 90-day mortality was 0.71 (95 % CI: 0.66–0.81), with an optimal cutoff value of 257 ng/ml [41].

In an observational retrospective study, plasma NGAL (AUC = 0.69) predicted the mortality in these sepsis patients within 7 days after admission to the ER, stronger than creatinine clearance (AUC = 0.61) but lower than the APACHE II score (AUC = 0.75) [37]. Likewise, the AUC of serum NGAL for predicting 28-day mortality in septic patients was 0.83 (95 % CI: 0.85–0.94) with a cutoff value of 236.62 ng/ml (sensitivity, 0.72; specificity, 0.77) [36].

Among 168 septic patients admitted to the ER, 72 % developed AKI. Urine NGAL during the first 24 hours after admission was a poor predictor of morbidity and mortality (AUC = 0.66 and 0.68, respectively), whereas urine NGAL (between 24 and 48 hours after admission) was a better predictor (AUC = 0.70 and 0.81, respectively) [32].

## Discussion

Sepsis is a major contributing factor to AKI in hospitalized patients, especially among those with critical illnesses [43, 44]. NGAL is among the most extensively researched biological markers for early prediction of AKI in both blood and urine specimens. Haase-Fielitz et al*.* [45] performed a systematic review and identified 58 articles that enrolled >16,500 patients, and found that both plasma and urine NGAL were predictive of AKI and its severity, with overall AUCs ranging from 0.79 to 0.87 in different clinical settings. However, it remains controversial whether NGAL is predictive of AKI in septic patients because of the lack of corresponding statistical data. The information on NGAL for prediction of RRT and mortality in AKI patients with sepsis was extremely limited. We systematically reviewed studies on the diagnostic accuracy of plasma and urine NGAL for prediction of AKI in septic patients. The pooled results indicated that plasma and urine NGAL showed good diagnostic precision of AKI with sepsis (AUC = 0.86 and 0.90, respectively). However, the systematic reviews of diagnostic accuracy studies were usually characterized by significant heterogeneity on account of the small sample sizes in most studies of plasma NGAL. Moreover, meta-regression, subgroup analysis, and publication bias were not performed to identify the sources of heterogeneity, which made it difficult to interpret the funnel plots. Meanwhile, for urine NGAL, although the threshold effect, heterogeneity test, subgroup analysis, meta-regression, and publication bias were analyzed to identify potential influencing factors, the cause of nonthreshold effects that induce heterogeneity was not particularly clear, probably because the differences in specimen sampling time and cutoff values among these included studies might have affected the heterogeneity and further analysis could not be conducted because of the limitations of the primary studies. Moreover, in some useful studies 2 × 2 contingency table data could not be extracted, which also led to bias of the results.

In addition, we noticed some interesting results reported in recent articles. In AKI patients, Bagshaw et al*.* [46] conducted a comprehensive observational study to assess the prospective evaluation of NGAL in septic vs. nonseptic AKI. The AUC of plasma NGAL (≥280 ng/ml) for the diagnosis of septic vs. non-septic AKI was 0.77 (95 % CI: 0.63–0.90) with a sensitivity of 0.75 and specificity of 0.76, while that of urine NGAL (≥150 ng/mg creatinine) was 0.70 (95 % CI: 0.59–0.82) with a sensitivity of 0.69 and specificity of 0.60 for a diagnosis of septic AKI. The peak plasma NGAL showed fair discriminatory power for prediction of AKI progression (AUC = 0.71, 95 % CI: 0.55–0.88) and need for RRT (AUC = 0.78, 95 % CI: 0.61–0.95), while urine NGAL performed less well for prediction of AKI progression (AUC = 0.70, 95 % CI: 0.58–0.81) and need for RRT (AUC = 0.70, 95 % CI: 0.58–0.82), and peak urine NGAL (≥230 ng/mg creatinine) predicted AKI progression with a sensitivity of 0.78 and specificity of 0.81, which was better than compared with septic AKI (AUC = 0.82 vs. 0.59, respectively; *P* = 0.04). Peak plasma and urine NGAL alone had poor discriminatory power for prediction of in-hospital death (plasma NGAL: AUC = 0.69; 95 % CI: 0.48–0.74; urine NGAL: AUC = 0.62; 95 % CI: 0.49–0.76) [46]. The study indicated that septic AKI patients had higher detectable NGAL compared with nonseptic AKI patients, but whether there was a correlation between sepsis and NGAL was unclear. Some scholars studied the predictive value of NGAL for sepsis. The AUC of plasma NGAL was 0.51 (95 % CI 0.46–0.56), even differing from that of procalcitonin (0.67, 95 % CI 0.62–0.72, *P* <0.01) in a prospective observational study [47]. High plasma NGAL was also observed that could independently predict mortality (AUC = 0.64) and multiple organ dysfunction syndrome in severe sepsis and septic shock during ICU stay (hazard ratio = 2.13; 95 % CI: 1.08–4.20; *P* = 0.03 and hazard ratio = 1.90; 95 % CI: 1.01–3.55; *P* = 0.046, respectively) [48]. In several prospective studies, NGAL levels were significantly higher among septic patients than nonseptic subjects, but the precise influence of the diagnostic test characteristics remains unclear [33, 46, 49].

A prospective single-center cohort study that included 663 admissions to the ICU found that the AUC of peak plasma NGAL values for AKI was unaffected by the presence of sepsis (0.78, 95 % CI: 0.67–0.86 for sepsis vs. 0.76, 95 % CI: 0.72–0.79 for nonsepsis; *P* = 0.72) [34]. Meanwhile, during sepsis, plasma NGAL was moderately to strongly correlated with cytokine interleukin (IL)-6 in septic patients and animal models, which clarified that plasma NGAL might be involved in immune responses during inflammation, rather than only restricted to the diagnosis of AKI. Nevertheless, in sepsis with AKI, plasma NGAL and tumor necrosis factor alpha (TNFα) were already elevated at 6 hours without changes to serum creatinine and blood urea nitrogen, while IL-6 and IL-10 are increased only after 24 hours. This phenomenon indicated that the early increase of plasma NGAL during sepsis was not solely a result of inflammation and cytokine storm but rather results from early kidney damage, which revealed the predictive value of early diagnosis of sepsis AKI. The association of TNFα with NGAL showed that septic AKI might be mainly initiated by TNFα, also explaining why higher NGAL levels were found in septic vs. nonseptic AKI [50].

## Conclusion

Early and efficient diagnosis of AKI is of great significance to the prognosis of critically ill patients. In conclusion, to a certain extent, NGAL is not only an effective predictive factor for AKI in the process of sepsis, but also shows potential predictive value for RRT and mortality. The results of the current study motivated us to reconsider the value of NGAL for prediction of AKI in septic patients. More future trials with larger sample sizes and high-quality evidence are needed to clarify this controversial issue for further improvement of patient outcomes.

## Key messages

- AKI is a widespread problem in critically ill patients, and sepsis is a well-known precipitating factor for the development of AKI.
- NGAL is not only an effective predictive factor for AKI in the process of sepsis, but also shows potential value for RRT and mortality.

## Abbreviations

ACCP/SCCM: American College of Chest Physicians/Society of Critical Care Medicine
AKI: Acute kidney injury
AKIN: Acute Kidney Injury Network
APACHE: Acute Physiology and Chronic Health Evaluation
AUC: Area under the curve
CI: Confidence interval
DOR: Diagnostic odds ratio
ELISA: Enzyme-linked immunosorbent assay
ER: Emergency room
FN: False-negative
FP: False-positive
IL: Interleukin
KDIGO: Kidney Disease Improving Global Outcomes
NGAL: Neutrophil gelatinase-associated lipocalin
NLR: Negative likelihood ratio
PLR: Positive likelihood ratio
QUADAS-2: Quality Assessment of Diagnostic Accuracy Studies 2
RIFLE: Risk, Injury, Failure, Loss, End-stage Kidney Disease
RRT: Renal replacement therapy
SCCM/ESICM/ACCP/ATS/SIS: Society of Critical Care Medicine/European Society of Intensive Care Medicine/American College of Chest Physicians/American Thoracic Society/Surgical Infection Society
SROC: Summary receiver operating characteristic
TN: True-negative
TNFα: Tumor necrosis factor alpha
TP: True-positive
